# The Related Transcriptional Enhancer Factor-1 Isoform, TEAD4_216_, Can Repress Vascular Endothelial Growth Factor Expression in Mammalian Cells

**DOI:** 10.1371/journal.pone.0031260

**Published:** 2012-06-22

**Authors:** Binoy Appukuttan, Trevor J. McFarland, Andrew Stempel, Jean B. Kassem, Matthew Hartzell, Yi Zhang, Derek Bond, Kelsey West, Reid Wilson, Andrew Stout, Yuzhen Pan, Hoda Ilias, Kathryn Robertson, Michael L. Klein, David Wilson, Justine R. Smith, J. Timothy Stout

**Affiliations:** Casey Eye Institute, Oregon Health and Science University, Portland, Oregon, United States of America; International Centre for Genetic Engineering and Biotechnology, Italy

## Abstract

Increased cellular production of vascular endothelial growth factor (VEGF) is responsible for the development and progression of multiple cancers and other neovascular conditions, and therapies targeting post-translational VEGF products are used in the treatment of these diseases. Development of methods to control and modify the transcription of the VEGF gene is an alternative approach that may have therapeutic potential. We have previously shown that isoforms of the transcriptional enhancer factor 1-related (TEAD4) protein can enhance the production of VEGF. In this study we describe a new TEAD4 isoform, TEAD4_216_, which represses VEGF promoter activity. The TEAD4_216_ isoform inhibits human VEGF promoter activity and does not require the presence of the hypoxia responsive element (HRE), which is the sequence critical to hypoxia inducible factor (HIF)-mediated effects. The TEAD4_216_ protein is localized to the cytoplasm, whereas the enhancer isoforms are found within the nucleus. The TEAD4_216_ isoform can competitively repress the stimulatory activity of the TEAD4_434_ and TEAD4_148_ enhancers. Synthesis of the native VEGF_165_ protein and cellular proliferation is suppressed by the TEAD4_216_ isoform. Mutational analysis indicates that nuclear or cytoplasmic localization of any isoform determines whether it acts as an enhancer or repressor, respectively. The TEAD4_216_ isoform appears to inhibit VEGF production independently of the HRE required activity by HIF, suggesting that this alternatively spliced isoform of TEAD4 may provide a novel approach to treat VEGF-dependent diseases.

## Introduction

Alternate splicing to generate multiple mRNAs and subsequent protein products from one gene is exploited in many organisms, and in different tissues within the same organism, to augment the functional complexity of the translated genome. [1]–[3] Transcriptional enhancer factor 1 (TEF-1) is a member of the TEA DNA-binding family (TEAD), in which tissue and disease specific isoforms, generated by alternate splicing, have been observed. [4], [5] The TEAD family of proteins is remarkably conserved from yeast to humans and, depending upon interaction with other proteins, can either activate or repress gene expression. [6] Four TEAD genes exist in mammals (TEAD 1 to 4) and expression of these genes has been characterized in various mammalian tissues and cell types.[7]–[9] Transcriptional enhancer factor 1-related (RTEF-1 or TEAD4) protein was originally reported to regulate muscle-specific genes in cardiac and smooth muscle cells. [8] The TEAD4 transcription factor requires the presence of myocyte-specific (M-CAT) sequences and muscle-specific cofactors to facilitate cell-specific gene regulation. Human and mouse TEAD4 transcripts exhibit dissimilar tissue specific expression profiles, where highest expression in humans is observed in pancreas and skeletal muscle with low levels in heart and kidney, whereas murine lung tissue has the most abundant message with low levels in heart and skeletal muscle. [7], [10] The mouse embryo expresses high levels of TEAD4 message within skeletal muscle as well as an alternatively spliced isoform that lacks exon 5 relative to the full-length gene, indicating that within the mouse, TEAD4 is regulated developmentally and at least one isoform exists. [10] Alternative splicing of TEAD4 in human cells and regulation of non-muscle specific genes is less well characterized.

Recently, alternatively spliced transcripts for TEAD4 have been identified in human retinal vascular endothelial cells. [11] Shie and colleagues showed that full length TEAD4 protein was able to bind to Sp1 sequences within the VEGF promoter and enhance VEGF gene expression in bovine aortic endothelial cells. [12] TEAD4 expression was up-regulated under hypoxic conditions, and TEAD4 mediated stimulation of VEGF expression was independent of the classic hypoxia responsive element (HRE) and hypoxia-inducible factor (HIF-1) mechanism. [12] We identified two new human isoforms, 936 (TEAD4_311_) and 447 (TEAD4_148_), which enhanced VEGF promoter activity. The TEAD4_148_ isoform was the most potent enhancer relative to other isoforms. Interestingly, the TEAD4_148_ isoform was identified in human ocular endothelial cells cultured under hypoxic conditions, suggesting that environment-specific alternate splicing may occur within human tissue to generate specific transcription factors with altered functions. In this study, we report an alternatively spliced TEAD4 transcript that generates a novel isoform (TEAD4_216_) able to significantly repress promoter activity. This novel protein reduces native VEGF production and cell proliferation. We show that the TEAD4_216_ isoform can function in the absence of the HRE sequence and overcomes the enhancement mediated by the other TEAD4 isoforms. In addition we demonstrate the presence of TEAD4 protein in choroidal neovascular membrane in human age-related macular degeneration, suggesting a role in human disease.

## Results

### Sequence Comparison of TEAD4_216_ to Other TEAD4 Isoforms

**Figure 1:**
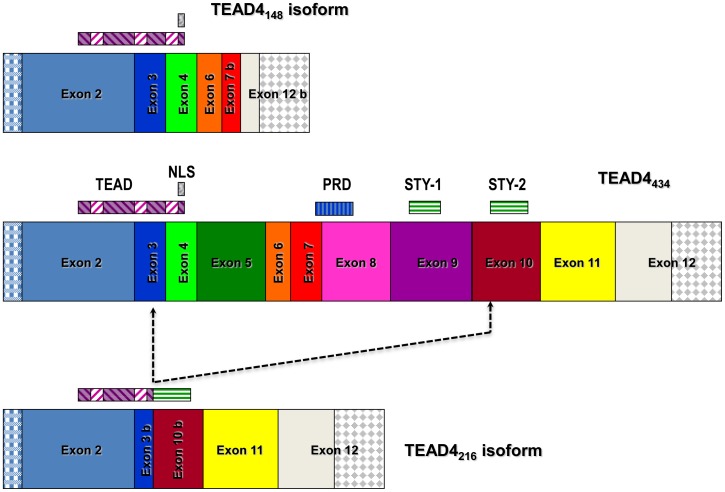
Schematic diagram of the full length TEAD4 (TEAD4_434_) and the novel TEAD4_216_ isoform. The previously identified TEAD4_148_ isoform is also shown for comparison. Schematic indicates the TEA DNA-binding domain (TEAD), a putative nuclear localization signal (NLS), a proline rich domain (PRD) and serine-threonine-tyrosine (STY) domains.

Amplification from cDNA prepared from pooled total RNA isolated from primary cultures of human choroidal (PCVEC), retinal (PRVEC) and iris (PIVEC) vascular endothelial cells, with primers to full length TEAD4, detected the previously described TEAD4_434_ and TEAD4_311_ isoforms as well as a novel 651 bp product (Figure S1A). Amplification of specific isoforms not only varied between REC, CEC and IEC, but also differed under hypoxic conditions (Figure S1B). The 651 bp product was consistently amplified in all donors and ECs tested in this study. In addition, TEAD_434_ and other isoforms were detected by western blot analysis of primate retinal-choroidal endothelial cells (Figure S2). The 651 bp (TEAD4_216_) isoform is generated by an inframe splice event within exons 3 and 10 that results in loss of the 3′ end of exon 3, complete deletion of exons 4 to 9, and absence of the 5′ end of exon 10 (Figure 1). The donor splice site occurs at the junction of Thr-92 and Arg-93, removing the last five codons of exon 3, and the acceptor site occurs between Gly-310 and Ser-311, eliminating the first eleven codons of exon 10. The splicing event results in removal of the third alpha helix and a nuclear localization signal (NLS), within the TEA DNA-binding domain, the proline rich domain (PRD) and a hydroxyl containing amino acid rich STY domain (Figure 1). The combination of specific protein motifs present in the TEAD4_216_ isoform is novel when compared with the previously identified TEAD4_311_ and TEAD4_148_ isoforms. [11].

### The TEAD4_216_ Isoform Represses VEGF Promoter Activity

**Figure 2:**
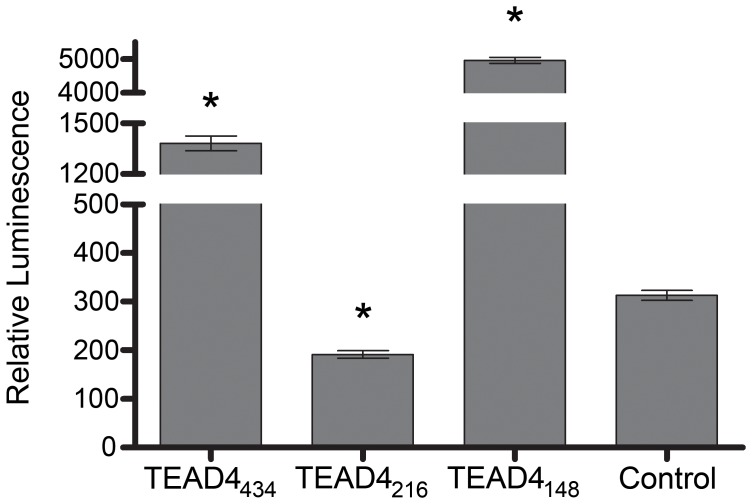
The TEAD4_216_ isoform represses VEGF promoter activity. The TEAD4_216_ isoform can repress expression from the full length human VEGF promoter F1–R3 (p<0.01, n = 9) in contrast to the TEAD4_434_ and TEAD4_148_ enhancers.

The full-length TEAD4 isoform (TEAD4_434_) acts as a transcriptional enhancer of VEGF in bovine aortic endothelial cells. [12] Reporter assays indicate that the TEAD4_311_ and TEAD4_148_ isoforms increase human VEGF promoter activity. [11] To test whether the TEAD4_216_ isoform was able to effect VEGF promoter-driven transcription, co-transfection experiments in 293T cells with a reporter plasmid that placed the secretable alkaline phosphatase (SEAP) gene under the transcriptional control of the VEGF promoter (1,136 bp of the 5′ proximal VEGF promoter and 54 bp of the VEGF gene 5′UTR), and an expression plasmid containing the TEAD4_216_ cDNA, were performed. The TEAD4_216_ isoform repressed VEGF promoter activity approximately 50% below background (P<0.05, Figure 2).

### The TEAD4_216_ Isoform Does not Require the HRE Sequence to Repress VEGF Promoter Activity

**Figure 3:**
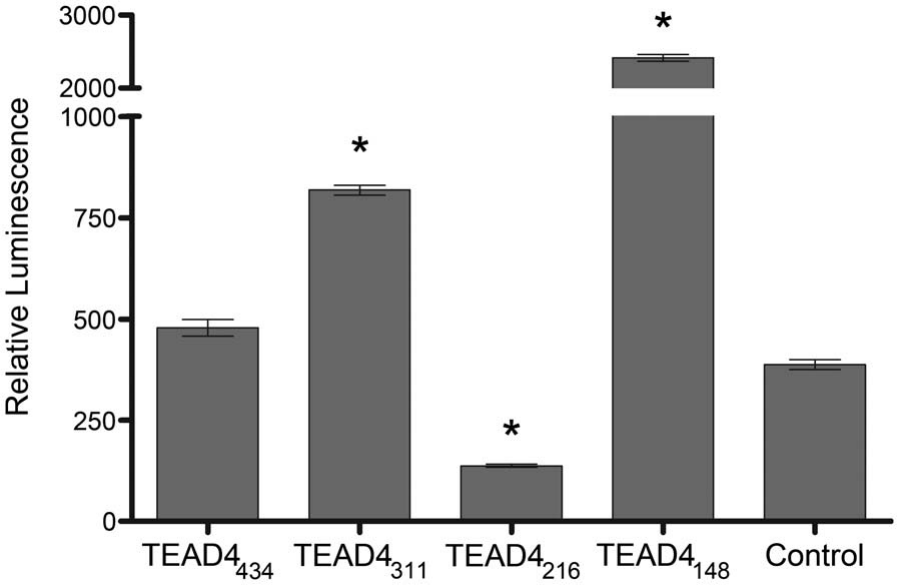
The TEAD4_216_ isoform does not require the HRE sequence to function. The TEAD4_216_ isoform can repress expression from the human VEGF promoter (F2–R3) that lacks the HRE (p<0.01, n = 6). The TEAD4_148_ and TEAD4_311_ enchancer isoforms do not require the HRE to promote reporter gene expression (p<0.001). However the full length TEAD4_434_ did not significantly enhance expression (p>0.02).

The hypoxia-inducible factor (HIF)-1 subunits require the presence of a specific sequence the HRE, situated between –985 and –939 bp within the human VEGF 5′ promoter to function as an enhancer of transcription. The HIF-1 induced VEGF expression is initiated under conditions of cellular oxygen starvation as observed in tumor tissue and ischemic disease.[13]–[16] Full-length human TEAD4 (TEAD4_434_) stimulates VEGF production independent of the HIF-1/HRE pathway in bovine endothelial cells. [12] To assess whether the TEAD4_216_ isoform requires the HRE sequence to repress VEGF activity, a reporter plasmid containing a truncated promoter fragment (lacking the HRE sequence) was generated, as described previously. [11] Using this plasmid, we demonstrated that the enhancer isoforms increased expression in 293T cells, whereas the TEAD4_216_ isoform retained its ability to inhibit VEGF promoter activity (Figure 3). The HRE-independent, TEAD4_216_-mediated repression of VEGF promoter activity was approximately 50% below background control levels. The full length TEAD4 requires four consecutive Sp1 sequences (Sp1–1 to Sp1–4) situated between −100 and −40 bp upstream of the human VEGF transcription initiation site to function as an enhancer. [11], [12] We show that TEAD4_216_ and TEAD4_148_ can also interact with the Sp1 sequences situated within the VEGF promoter (Figure S3, Methods S1).

### The TEAD4_216_ Isoform Competitively Inhibits VEGF Promoter Activity

**Figure 4:**
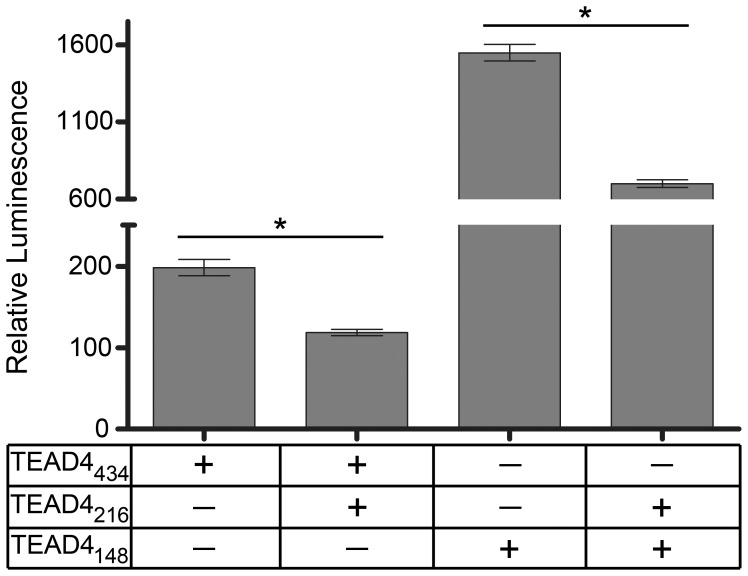
The TEAD4_216_ isoform can competitively inhibit promoter activity. The TEAD4_216_ isoform can competitively repress expression from the human VEGF promoter in the presence of either the TEAD4_434_ or TEAD4_148_ enhancer isoforms (p<0.005, n = 6).

Cotransfection experiments were performed to test whether the TEAD4_216_ isoform competitively inhibited VEGF promoter-directed transcription in the presence of stimulating isoforms. Equal copy numbers of TEAD4_216_ expression plasmid and one enhancer isoform were co-transfected into 293T cells with a pSEAP reporter vector under the control of the human VEGF promoter. The TEAD4_216_ isoform was able to significantly inhibit VEGF promoter activity in the presence of stimulatory isoforms (Figure 4). A dose response trend was observed in experiments that included the most potent stimulatory isoform (TEAD4_148_) (Figure S4). To ensure that expression plasmids containing each of the TEAD4 isoforms were capable of expressing protein and not degraded within the time frame of the transfection assays, a western blot confirmed presence of TEAD4_434_, TEAD4_216_ and TEAD4_447_ (Figure S5).

### The TEAD4_216_ Isoform Represses VEGF Promoter Activity Under Hypoxic Conditions

**Figure 5:**
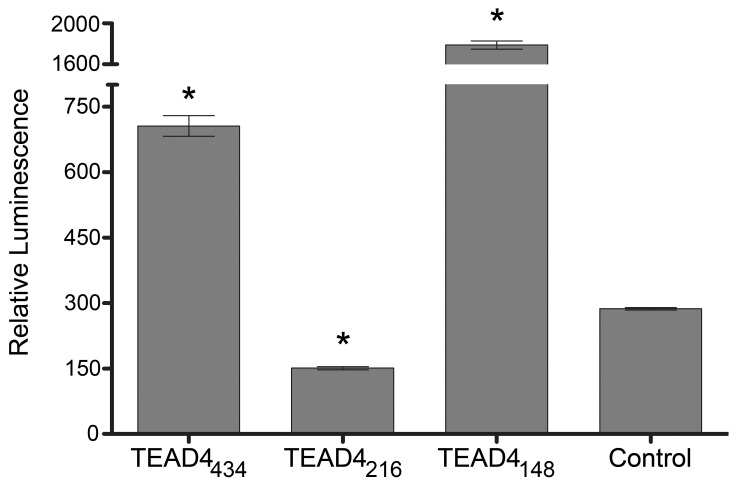
The TEAD4_216_ isoform can inhibit promoter activity under hypoxic conditions. The TEAD4_216_ isoform can still repress expression mediated from the human VEGF promoter (F1–R3) that includes the HRE sequence under conditions of hypoxia (p<0.001, n = 6).

Under hypoxic conditions, HIF-1 subunits bind to the HRE within the VEGF promoter to enhance gene expression. To test whether the TEAD4_216_ isoform represses promoter activity in a low oxygen environment, 293T cells were co-transfected with the pSEAP vector containing the human VEGF promoter and an expression plasmid containing one of each of the TEAD4 isoforms. Transfected cells were then cultured under hypoxic conditions. The TEAD4_216_ isoform repressed expression in the hypoxic environment suggesting inhibitory effect in pathophysiologically relevant conditions (Figure 5).

### The TEAD4_216_ Isoform Decreases Native VEGF Production and Inhibits Cell Proliferation

**Figure 6:**
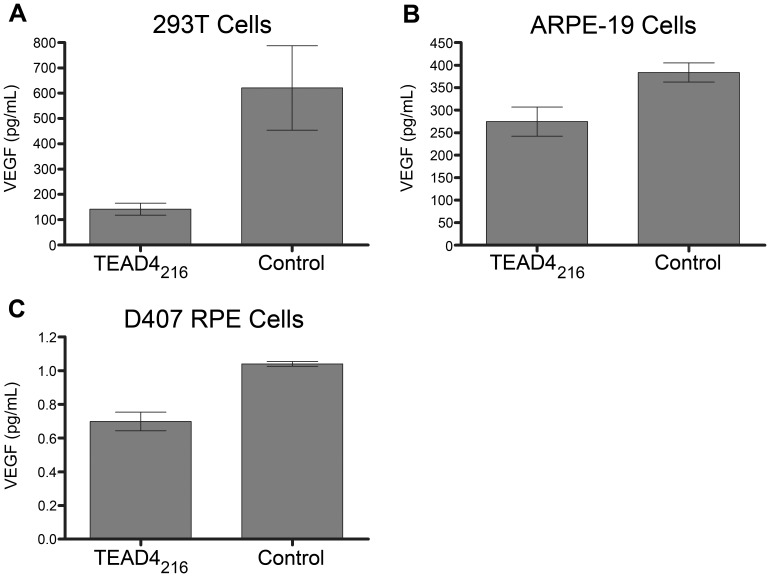
The TEAD4_216_ isoform can reduce endogenous VEGF_165_ protein. Transient plasmid transfection or stable lentiviral mediated introduction of TEAD4_216_ into human cells results in reduction of native VEGF protein. Solid bars represent VEGF_165_ levels, quantified by ELISA, within conditioned media collected 48 hours after transfection of pcDNA plasmid vector containing the TEAD4_216_ isoform into (**A**) 293T cells, (**B**) ARPE-19 retinal pigment epithelial (RPE) cells in culture (n = 4), (p<0.05). (**C**) Lentiviral (LV) expression of the TEAD4_216_ isoform in human D407 RPE cells can inhibit endogenous VEGF production. Solid bars represent VEGF_165_ levels, quantified by ELISA, within conditioned media collected 48 hours after transduced RPE cells were FAC sorted and plated (n = 3),(p<0.02).

**Figure 7:**
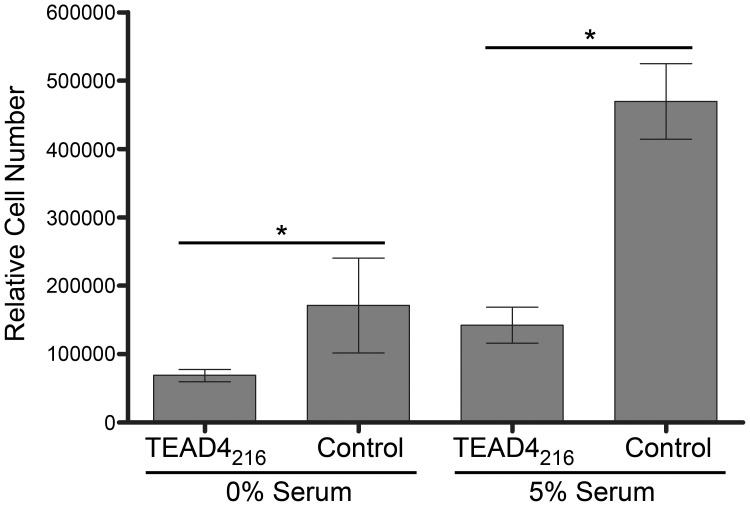
Lentiviral (LV) mediated expression of the TEAD4_216_ isoform in 293T cells inhibits cell proliferation. A Cyquant cell proliferation assay determining the DNA content of cells indicate that 4 days after initial seeding of equal cell numbers the control untransduced cells proliferate at a faster rate than the LV-TEAD4_216_ transduced cells (n = 8, p<0.05).

The 165 amino acid isoform of VEGF (VEGF_165_) is considered a key protein in the development of neovascular disease and neoplasia. [17] To test whether expression of the TEAD4_216_ isoform inhibits native VEGF production, cells were transfected with expression plasmids containing the TEAD4_216_ gene, and secreted VEGF_165_ was determined 48 hours following transfection by ELISA. Relative to the control, the TEAD4_216_ treated cells produced less VEGF protein (Figure 6). This reduction in native VEGF expression was observed in both 293T (Figure 6A) and retinal pigment epithelial (RPE) cells (Figure 6B). Lentiviral-mediated expression of the TEAD4_216_ isoform within RPE cells similarly reduced VEGF production (Figure 6C). A proliferation assay was performed to determine whether lentiviral mediated expression of the TEAD4_216_ isoform inhibits cell proliferation. Cells transduced with either a lentiviral vector containing the TEAD4_216_ isoform and GFP or GFP alone were FAC sorted and seeded at equal density into 96 well plates. Cells were cultured for four days in the presence or absence of serum and subsequently assayed for cell number. Cells expressing the TEAD4_216_ isoform proliferated at reduced rates regardless of the presence of serum (Figure 7).

### The TEAD4_216_ Transcription Factor is Localized to the Cytoplasmic Region

**Figure 8:**
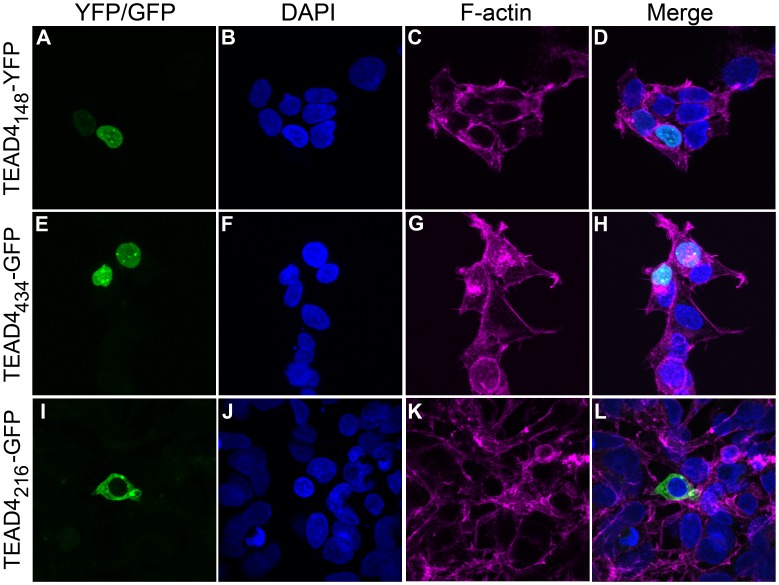
The TEAD4_148_ and TEAD4_434_ isoforms that enhance VEGF expression are localized to the nucleus whereas the TEAD4_216_ repressor isoform is maintained in the cytoplasm. TEAD4 isoforms were expressed as fusion proteins within 293T cells with various fluorescent proteins (FP) (TEAD4_434_ -Green-FP, and TEAD4_148_-Yellow-FP (pseudo-colored green) and TEAD4_216_-Green-FP) to visualize cellular localization.

The TEAD4_216_ isoform lacks the putative nuclear localization sequence (NLS) situated at the end of the TEA DNA-binding domain (TEAD) (Figure 1). Enhancer isoforms contain the complete TEAD and are assumed to function within the nucleus by binding to *cis*-elements within chromosomal DNA. In the case of the VEGF promoter, the full-length TEAD4_434_ transcription factor binds to Sp1 elements situated within 100 bp upstream of the transcriptional start site. [12] To determine the cellular localization of the TEAD4_148_, TEAD4_216_ and TEAD4_434_ proteins, fusion constructs of each isoform linked to fluorescent tags were prepared and expressed within mammalian cells. Fluorescent imaging demonstrated that the enhancer isoforms were located within the nucleus whereas the TEAD4_216_ isoform was present in the cytoplasm (Figure 8). It would appear that the NLS is essential for nuclear positioning of TEAD4 isoforms and may determine whether the protein acts as an enhancer or repressor.

### The TEAD4_216_ Gains Enhancer Activity with a Nuclear Localization Sequence

In order to dissect which domains within the TEAD4_216_ isoform affords repressor activity, mutant and chimeric constructs were generated (Methods S1) and their effect on the human VEGF promoter was analyzed (Figure S6). The repressor TEAD4_216_ containing the complete DNA binding domain including the NLS sequence (TEAD4_216_+NLS) enhanced promoter activity whereas the TEAD4_148_ without a NLS motif (TEAD4_148_-NLS) lost the ability to enhance. A chimeric containing the DNA binding portion of TEAD4_216_, lacking the NLS, and the non-DNA binding part of TEAD4_148_ (5′- TEAD4_216_+3′- TEAD4_148_), as well as the mutant isoform that consisted of the TEA DNA binding domain only (DNA BD, containing exons 2 to 4 of TEAD4_434_) also failed to enhance. Although, the combination of alternatively spliced sequences downstream of the complete TEA DNA binding domain may influence potency of enhancement, as evidenced by the TEAD4_434_ and TEAD4_216_+NLS isoforms, the same sequence loses enhancer function without a functional NLS (Figure S6).

### TEAD4 is Present in Ocular Tissue in Primate Ischemic or Neovascular Eye Disease

**Figure 9:**
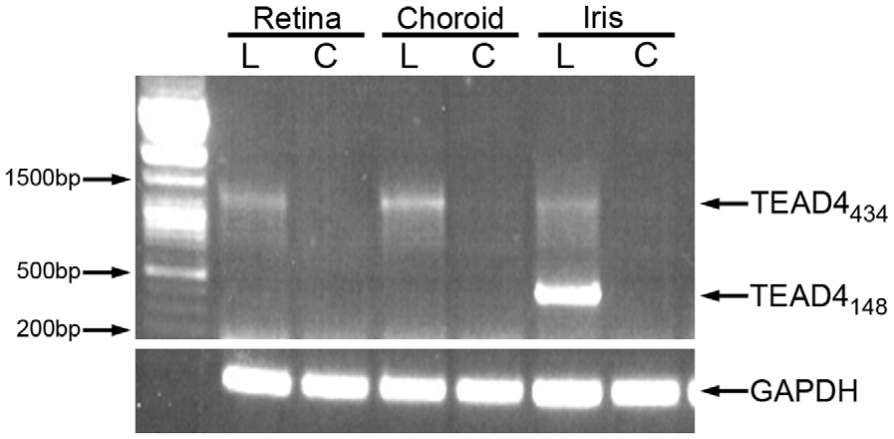
TEAD4 is upregulated and alternatively spliced within the eye in an animal model of ocular ischemic disease. RT-PCR for TEAD4 from choroidal, retinal and iris tissue isolated from a non-human primate eye (*Rhesus macaque*) 24 hrs after occlusion of the central retinal artery (CRAO), indicates that the full length TEAD4_434_ transcript is increased and the TEAD4_148_ enhancer isoform is produced in the lasered eye. **L** =  Lasered CRAO eye; **C**  =  control eye.

Occlusion of the central retinal artery or vein leads to ocular hypoxia and often results in a rapid increase of intra-ocular VEGF. [18], [19] Analysis of retinal, choroidal and iris tissue for TEAD4 RNA in a non-human primate (NHP) model of central retinal artery occlusion (CRAO) indicated that the TEAD4_434_ form of TEAD4 was upregulated in the ischemic eye and undetectable in the contralateral control eye (Figure 9). In addition, the NHP orthologue of the human TEAD4_148_ isoform was found to be present in iris tissue from the ischemic eye and not in the normal control eye (Figure 9). Sequence comparison of the human TEAD4_148_ and NHP TEAD4_148_ isoforms indicated 98.4% identity at the nucleotide and 96.6% identity at the protein level.

**Figure 10:**
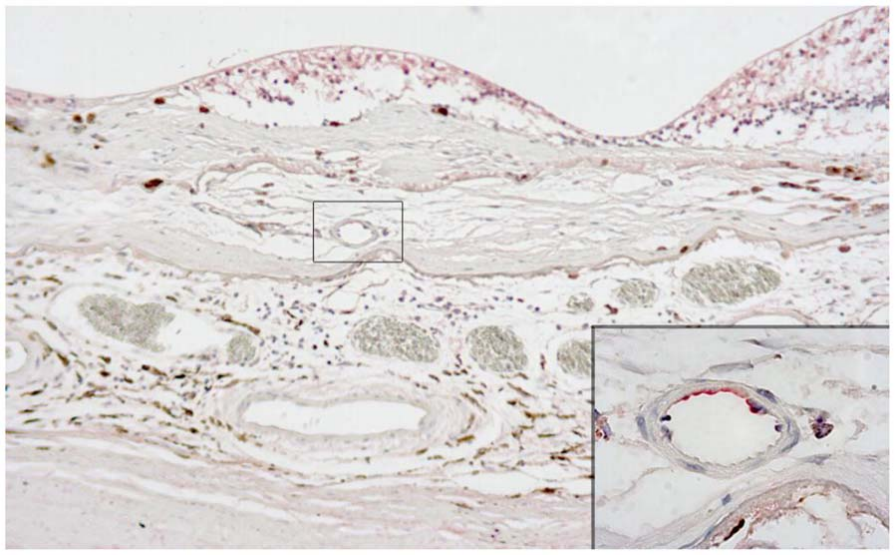
Human TEAD4 protein is present on new vessels in neovascular AMD lesion. Photomicrograph showing a subretinal neovascular membrane in a human ocular tissue section (Fast Red, TEAD4; hematoxylin counterstain; original magnification x 100). Insert (location indicated by rectangle; original magnification x 1000) shows positive staining for TEAD4 by vascular endothelium of a choroidal new vessel that has bridged the elastic lamina of Bruch’s membrane.

Choroidal neovascularization is a common sequelae of age-related macular degeneration and is a VEGF-mediated process. [20], [21] To test whether TEAD4 is present in neovascular complexes associated with AMD, antibodies raised against human TEAD4 were used in immunohistopathologic analysis of sections from eyes with neovascular AMD. Hematoxylin and eosin staining demonstrated subretinal neovascular membranes in the three globes obtained post-mortem from three patients with age-related macular degeneration. Endothelium of choroidal new blood vessels within the membrane stained positively for TEAD4 in two eyes (Figure 10); the third eye showed relatively weak staining overall. In addition and for all eyes, positive extracellular staining was observed within the membrane. This finding was attributed to leakage of serum from the abnormal vasculature into the tissue, since similar staining of serum was also present within vessels. Subretinal blood stained positively in one eye. In addition to staining within the neovascular membrane, anti-TEAD4 antibody recognized some other ocular cells. Positive staining was identified in retinal pigment epithelium and corneal epithelium, and for one globe that showed most staining overall, there was also convincing staining of ciliary epithelium, retinal neurons and endothelium of some normal vessels in the choroid and pars plana. Tissue sections stained with rabbit IgG revealed no positive staining.

## Discussion

We report the discovery of a novel TEAD4 isoform, isolated from vascular endothelial cells derived from human eyes, that represses gene expression from the VEGF promoter. We note that through alternative splicing of TEAD4 transcripts, different proteins are synthesized; some of these isoforms act as strong enhancers of transcription from the VEGF promoter, while others act as strong repressors.

Vascular endothelial growth factor has been implicated as a key component responsible for the onset and promotion of neovascularization. VEGF-associated neovascularization is involved in the pathology of blinding eye diseases such as proliferative diabetic retinopathy, retinopathy of prematurity (ROP) and AMD. In addition to ocular disease, solid tumor growth requires abundant neovascularization to supply nutrients for survival and growth. Thus, it is not surprising that drugs that inhibit the actions of VEGF proteins are useful in the treatment of ocular neovascular disease and neoplasia. [22], [23] On the other hand, the protein factors that control VEGF transcription in health or disease is less understood; these proteins may also prove to be potential therapeutic targets.

Under hypoxic conditions, expression of VEGF is upregulated in a variety of tissues. This hypoxia-mediated stimulation of VEGF is attributed mainly to the stabilization of the transcription factor HIF-1 alpha. [13], [24] Targeting HIF-1 alpha may be useful in inhibiting neovascular disease, but as with most biological systems, alternative mechanisms and pathways provide functional redundancy. Recently, the full length TEAD4 protein (TEAD4_434_) was shown to stimulate VEGF gene transcription under hypoxic conditions in bovine cells and to act independently of the HIF-1 pathway. From hypoxic ocular endothelial cells, we recently identified an alternatively spliced TEAD4 transcript able to encode a new isoform (TEAD4_148_) that is capable of enhancing VEGF promoter activity 3 to 5 times greater than that achieved by the full length TEAD4 protein and 10 times greater than the control. Whether specific alternatively spliced products of TEAD4 are biologically relevant to VEGF-dependent diseases is unknown. Nevertheless, disease specific splicing of TEAD4 within the mouse retina in a model of ROP has been noted. [11] Disease specific splicing within human tissue of other members of the TEAD family has also been observed. Whether repressing the transcription-enhancing isoforms or employing the transcription-inhibiting isoforms can help disrupt neovascularization remains to be determined.

The initial discovery of VEGF as a secreted molecule was observed in rodent and human tumor cell lines, and the protein was believed to be a mitogen specific to endothelial cells.[25]–[28] Subsequently, VEGF was shown to act as a potent inducer of cell proliferation, migration or survival in a wide variety of cells not of endothelial origin, including smooth muscle cells, retinal cells and neurons, glial cells, pericytes and mammary tumor cells.[29]–[40] Inhibition of VEGF production in tumor cells may help in treatments for cancer. We demonstrate that lentiviral transduction of TEAD4_216_ into HEK 293T cells results in an inhibition of proliferation in both serum-containing and serum-free media as measured by a cell proliferation assay. The ability of the TEAD4_216_ isoform to act as a suppressor of cell division is intriguing and may prove to be of therapeutic benefit.

We have previously demonstrated that TEAD4 transcripts are alternatively spliced within the retina of a mouse model of ROP. [11] We now show that the full length TEAD4_434_ is upregulated under ischemic conditions within the primate eye. Interestingly, in a primate CRAO model, expression of the TEAD4 isoform with highest stimulatory activity (i.e. TEAD4_148_) is seen at highest levels in iris tissue, a site of clinically relevant neovascularization in humans with vaso-occlusive disease. [41] Neovascularization of the iris is noted as a consequence of blockage of retinal blood vessels in primates and VEGF is known to play a significant role. [42], [43] The VEGF receptors, VEGFR1 and VEGFR2, and VEGF are expressed within primate iris tissue. [44] It is possible that the TEAD4_148_ isoform may be produced within the iris under conditions of ocular hypoxia to initiate the VEGF cascade thereby stimulating neovascularization. Thus, it would appear that not only expression, but also alternative splicing of the TEAD4 gene, is regulated by hypoxia and this mechanism may be tissue and/or cell specific (Figure S1).

Vascular endothelial growth factor is a key component to the etiology of AMD and treatments for blocking the action of VEGF have proven to be standard of care for this disease. [20] We have shown that human choroidal endothelial cells express TEAD4 transcripts *in vitro* and that expression is upregulated under conditions known to stimulate neovascularization. The potential role of hypoxia in the stimulation of VEGF expression in AMD is unclear. Here, we demonstrate the presence of TEAD4 protein in choroidal neovascular complexes in human eyes with neovascular AMD (Figure 10). Interestingly, HIF is absent from choroidal endothelial cells in AMD-related neovascular complexes, and thus a HIF-independent, TEAD4 pathway may regulate VEGF expression in these cells. [45].

Analysis of the TEAD4_216_ isoform suggests that it acts as an inhibitor of transcription of the VEGF gene even in the presence of stimulatory isoforms (Figure 4). Sequence analysis of the TEAD4_216_ isoform shows that it lacks a portion of the TEA DNA binding domain (the third alpha helix which includes a nuclear localization signal) that is retained by enhancer isoforms. Fusion protein experiments indicate that the TEAD4_216_ isoform remains in the cytoplasm, while the enhancing isoforms are found within the nucleus; suggesting that the repression effect may occur within the cytoplasm. It is possible that TEAD4_216_ mediated inhibition of VEGF occurs by some other indirect pathway, that involves protein degradation or regulation of other genes, that maybe cell- and cell-state specific. [46] Understanding the mechanism of action of this promoter repressor may lead to the discovery of essential cofactors that are required for the function of TEAD4. [47], [48] How competitive inhibition is achieved when the enhancer isoforms are physically located within the nucleus is intriguing and warrants further investigation. Most recently, specific cofactors that are essential for TEAD4_434_ mediated induction of the VEGF gene in muscle cells and that TEAD4_434_ may also upregulate the HIF-1 alpha gene via MCAT sequences in endothelial cells have been noted. [49], [50] Whether the other TEAD4 isoforms possess the ability to effect expression of the HIF-1 alpha gene or require the same specific cofactors for function and if this is a cell specific phenomenon needs further study. The full length TEAD4_434_ interacts with YAP65 to mediate transcription from muscle-specific genes. [51] Introduction of TEAD4 into cells lacking all TEAD proteins failed to elicit gene transcription until YAP was cointroduced with a TEAD protein. [51] The carboxyl half of the TEAD4 protein, amino acids 224 to 434, is considered essential for binding to YAP and more specifically four specific amino acids, K297, W299, F337 and Y422 are critical for YAP interaction. [51], [52] The amino acids K297 and W299 are within exon 9, F337 is contained in exon 10 while Y422 is in exon 12. Only TEAD4_434_ contains all 4 of these amino acids, whereas the repressor TEAD4_216_ possesses F337 and Y422 only, while the potent enhancer TEAD4_4148_ contains Y422 only (Figure 1). Thus, it is unlikely that either TEAD4_216_ or TEAD4_148_ binds YAP with strong affinity and may use other cofactors to mediate their function.

Other cofactors such as transcriptional coactivator with PDZ binding motif (TAZ), interferon response factor 2 binding protein 2 (IRF2BP2), a basic helix-loolp-helix leucine zipper protein (MAX), and serum response factor (SRF) a member of the MADS box superfamily of transcription factors have all been implicated as potential cofactors of TEAD4 and other members of the TEAD family of proteins. [47], [48], [50], [53] Interestingly, both TAZ and YAP are involved in the Hippo signaling pathway, an organ growth control system that involves the regulation of genes responsible for cell proliferation and apoptosis.[52], [54]–[56] The TAZ protein is a 14-3-3 binding protein and both YAP and TAZ activity are repressed by the Hippo pathway, by phosphorylation and sequestering of these proteins to the cytoplasm. Perturbation of the Hippo pathway has been implicated in cancer and uncontrolled cell division. [57] Considering that TEAD is essential for YAP-mediated cell growth, it is not unreasonable to consider the possibility that inhibitor TEAD4_216_ isoform may play a role or be utilized to regulate the Hippo pathway.

Characterizing the function of the new TEAD4_216_ isoform may lead to a better understanding of the control of VEGF gene regulation and may allow the development of therapeutics to inhibit VEGF gene transcription, pathologic neovascularization and cell proliferation through a HIF-independent mechanism.

## Materials and Methods

### Cultured Cells

Primary human ocular vascular endothelial cells were isolated from human retinal, iris and choroidal tissue and cultured as previously described, in the laboratory of Dr James T. Rosenbaum. [11] Total RNA was extracted from these cells when they were 99% pure, as evaluated by morphologic criteria, and at passages 2 to 5. Human 239T cells and ARPE-19 cells were purchased from ATCC and cultured in DMEM with 10% FBS, or serum free, and used for promoter reporter assays. The D407 RPE cell line (a gift from Dr. R. C. Hunt, University of South Carolina, Columbia, SC) were cultured in RPMI media with 10% FBS.

### Induction of Hypoxia

Cells were cultured in either 6 well or 12 well plates after transfection with expression and reporter gene plasmids. Cells were recovered overnight under normoxic conditions, and media was exchanged before placement in the Modulator Incubator Chamber (Billups-Rothenberg, Del Mar, CA) for hypoxic gas flushing. A 1% O_2_, 5% CO_2_ and 94% N_2_ gas mix was flushed through the chamber for exactly 5 minutes. The chamber was sealed and placed in a humidified 37°C incubator. After an 8 hour incubation, the chamber was flushed again with hypoxic gas for 5 minutes, and resealed and incubated for a further 8 hours. The flush and reincubation sequence was repeated once more before total RNA was isolated. Media was collected for reporter protein analysis 8 hours after the final flush.

### RNA Extraction and RT-PCR

Total RNA was isolated using an RNAqueous kit (Ambion Inc, Austin, TX) and 150 ng of this RNA was used with an oligo-dT primer for first strand synthesis (SuperScript II, Stratagene, La Jolla, CA). The following primers, F1: 5′-ttggagggcacggccggca-3′ and R1: 5′-tcattctttcaccagcctgta-3′ was used for second strand PCR amplification of TEAD4 using standard conditions. Amplified products were electrophoresed and visualized on a 1.5% agarose gel and subsequently purified from the gel (Qiaquick Gel Extraction, Qiagen, Valencia, CA) for standard dideoxynucleotide sequencing on an ABI 310 automated sequencer.

### Reporter Gene Analysis

The cDNA for TEAD4 isoforms were directionally cloned into the pcDNA 3.1 expression plasmid (Invitrogen, Carlsbad, CA). Human VEGF 5′ proximal promoter fragment (F1–R3) of 1,136 bp containing 54 bp of 5 UTR and 1,082 bp upstream of the transcription start site (TSS) was cloned 5′ to the secretable alkaline phosphatase (SEAP) gene within the pSEAP reporter plasmid (Clontech, Mountain View, CA). Truncated VEGF promoter fragment (F2–R3), which lacked the HRE sequence, containing 580 bp sequence 5′ to the TSS, was generated by nested amplification from the F1–R3 clone. The F2–R3 fragment was directionally cloned into the promoterless pSEAP vector. All constructs were sequence confirmed on both strands prior to transfection studies.

### Transfection Assays

The Amaxa Nucleofection Device (Amaxa Inc, Gaithersburg, MD), with Amaxa reagents and standard manufacturer’s protocol, was used to transfect plasmid vectors into cells. Briefly, 293T cells were cultured until 80% confluent and subsequently trypsinized. One million cells were resuspended in 100 µl of Nucleofect solution, mixed with 5 µl (containing 2 µg) of total plasmid DNA, and then electroporated (program #A023). Nucleofected cells were immediately resuspended in pre-warmed media and seeded into single wells of a 6 or 12-well plate. Cells were recovered overnight, before media was replaced with exactly 500 µl of fresh media. After 6 to 24 hours incubation, 200 µl of media was carefully removed and 25 µl of this was assayed immediately or stored at −20°C for SEAP analysis. Two separate 25 µl media aliquots were used for SEAP analysis according to manufacturer’s protocol (BD Biosciences, San Jose, CA). The two readings from one well were averaged for comparison to triplicate/quadruplicate repeat wells. Each co-transfection was repeated in triplicate or quadruplicate for a single experiment. Each experiment was repeated independently two more times with separate plasmid preparations (n = 9–12). A representative experiment is presented. Statistical analysis was performed using a Student’s t-test (two-tailed) to compare the triplicate samples in a single experiment. Bonferroni correction for multiple testing was applied and a P<0.01 was considered as significant.

For each co-transfection, the copy number of each plasmid was adjusted to be equivalent to the copy number of the largest plasmid used. The pcDNA 3.1 expression plasmid with no insert served as a control. As positive controls, a SV40 promoter pSEAP plasmid and a pGFPmax vector were transfected to ensure efficient and equal transfection efficiencies. The pSEAP plasmid with an SV40 promoter served as a positive control for subsequent SEAP protein analysis. The pGFPmax vector also served as positive control for transfection for each batch of cells allowing visual confirmation of consistent transfection efficiency. Nucleofection consistently gave 75–90% transfection efficiency in all experiments.

### Quantification of Secreted VEGF Protein

The level of VEGF_165_ protein was quantified using a standard ELISA kit (Chemicon, Temecula, CA), according to the manufacturer’s protocol. Cells were Nucleofected as described above with either 2 µg of pcDNA vector containing the cDNA for TEAD4_216_ or an equal copy number of the vector with no insert as a control. Cells were recovered overnight, and the media was replaced. The conditioned media was collected 24 hrs later and 2×50 µl of media was collected from each well (n = 4) for use in the ELISA.

### Laser Induced Central Retinal Artery Occlusion (CRAO)

All animal experiments were approved by the Institutional Animal Care and Use Committee and handled in accordance with the ARVO statement for the Use of Animals in Ophthalmic and Vision Research. Retinal artery occlusion was induced in the right eye of a cynomolgus primate via Rose bengal-assisted laser occlusion technique (532 nm laser); the left eye served as a control. Color photographs and fluorescein angiography (FA) were taken immediately after the procedure to confirm occlusion after the CRAO was induced. Briefly, cynomolgus primates (*Macaca fascicularis*, Sierra Biomedical Inc., Whitmore CA) were anesthetized by intramuscular injection of a mixture containing ketamine hydrochloride (20 mg/ml) and acepromazine maleate (0.125 mg/ml). Pupils were dilated (1% Cyclogel, 2.5% Phenylepherine, 0.5% Tropicamide). Eyes were topically sterilized and proparacaine hydrochloride was used as topical anesthesia. Immediately after Rose Bengal (Sigma, St. Louis, Mo, 20 mg/ml in normal saline) was injected into the femoral vein (20 mg/kg), the central retinal artery was irradiated with a 532 nm argon green laser (Iridex Inc., Mountain View, CA) via a stabilized slit-lamp delivery system. Following color photography (Ziess, fundus camera), 0.5 ml of fluorescein was injected and FA was performed to confirm occlusion of the central retinal artery. Retinal, choroidal, and iris tissue were harvested 24 hours after occlusion, and RNA was obtained. Total RNA was isolated from the three tissue groups (Ambion), quantified and followed by semi-quantitative RT-PCR for the TEAD4 transcript. The GAPDH housekeeping gene served as the loading control.

### Cell Proliferation Assay

Cell proliferation was determined using a standard assay (CyQuant NF Cell Proliferation Assay Kit, Molecular Probes, Eugene OR). The TEAD4_216_ cDNA insert was cloned into the pLenti7.3 vector (Invitrogen, Carlsbad, CA) and lentivirus (LV-TEAD4_216_) was prepared according to the manufacturer’s protocol. The LV-TEAD4_216_ virus was used to transduce 293T cells in wells of a 6-well plate, and transduced cells were FAC sorted using the emerald GFP encoded by the vector. LV-GFP was used as a control. LV-TEAD4_216_ positive cells were seeded at 10^5^ cells in 80 µl of media into each well of a 96-well culture plate (N = 8). Cells were incubated for 3 to 4 days at 37°C with 5% CO_2_ in a humidified chamber. Media was carefully aspirated from wells without disturbing the cells. Exactly 50 µl of Cyquant NF staining solution was added to each well, and wells were incubated at 37°C with 5% CO_2_ for 30 min to 1 hour. The plate was read in a Victor^3^ plate reader (Perkin Elmer, Waltham, MA). Statistical significance was determined by a paired Student’s *t*-test. Each experiment was performed in octuplicate and each proliferation assay was performed at least twice.

### Immunostaining of Eyes with Neovascular AMD

Three globes from 3 patients diagnosed with AMD were obtained post-mortem, fixed in 10% neutral buffered formalin and embedded in paraffin; these individuals were aged 77, 80 and 85 years at time of death. The Oregon Health & Science University Institutional Review Board provided a determination that this study did not constitute human subjects research. Ocular cross-sections were cut 5 microns thick, and mounted on Superfrost Plus glass slides (Fisher Scientific, Pittsburgh, PA) and heated overnight at 37°C, followed by 1 hour at 60°C, to promote firm adhesion.

TEAD4 was visualized in tissue sections, according to a previously published indirect immunostaining method [58], using rabbit polyclonal anti-TEAD4 antibody (made to order by Genemed Synthesis, San Antonio, TX, see Methods S1 for details of custom made antibody) or, as negative control, rabbit IgG (Vector Laboratories, Burlingame, CA), at a concentration of 11 ug/mL. Antigen retrieval was performed by immersing the slides in 10 mM citrate buffer (pH 6.0) and boiling for 10 minutes in a microwave oven. To visualize antibody complexes, sections were incubated with Fast Red (Biogenex, San Ramon, CA), as described by the manufacturer. Subsequently the sections were counterstained with hematoxylin. Immunostaining was interpreted by comparison with hematoxylin and eosin-stained sections.

## Supporting Information

Figure S1 **Human TEAD4 is differentially spliced within retinal, choroidal and iris derived vascular endothelial cells under hypoxia/normoxia.** Agarose gel electrophoresis showing RT-PCR from RNA isolated from primary cultures of REC, CEC and IECs after culture under normoxic or hypoxic conditions. Donor #1 (pooled REC, CEC and IEC cells) gave products for TEAD4_434_ (1305 bp band), TEAD4_311_ (936 bp band), TEAD4_216_ (651 bp band) and TEAD4_148_ (447 bp band) under hypoxic conditions, and under normoxic conditions only the TEAD4_216_ product was amplified. TEAD4_216_ was present in REC, CEC and IEC but interestingly expression levels differed between each EC type and between normoxic and hypoxic conditions.
(TIF)

Figure S2 **Western blot for TEAD4 on nonhuman primate ocular vascular endothelial cell proteins recognizes TEAD4_434_ and other isoforms.** Two different commercially available antibodies (ARP33426 and ARP38276, Aviva) recognize the full length TEAD4 (∼50 kD) in monkey retinal-choroidal ECs (RF/6A). No significant difference in TEAD4 expression was observed when normoxic (N) samples were compared to hypoxia (H) treated samples. Other TEAD4 isoforms appear to also be recognized at 30 KD, 26 kD and 24 kD.
(TIF)

Figure S3 **Band shift (EMSA) assay for TEAD4 isoforms on Sp-I sequences found within the VEGF promoter.** The enhancer isoforms TEAD4_434_, TEAD4_216_ and TEAD4_148_ bound to the SpI sequences present within the human VEGF promoter. The repressor isoform TEAD4_216,_ although lacking one of the 3 helixes present within the TEA DNA binding domain, is still able to bind the Sp-I sequences within the VEGF promoter.
(TIF)

Figure S4 **TEAD4_216_ can competitively inhibit enhancer mediated VEGF promoter activity.** Increasing concentrations of a plasmid containing TEAD4_216_, inhibited the enhancer properties of TEAD4_148_.
(TIF)

Figure S5 **Expressed protein from transfected TEAD4 isoforms is stable over time.** Protein from cells was isolated at various time points (24 to 96 hours) after transfection with pcDNA expression vector containing a TEAD4 isoform. Western blot was performed with a custom made TEAD4 antibody (Genemed Synthesis).
(TIF)

Figure S6 **TEAD4_216_ requires an NLS to enhance VEGF promoter activity.** A reporter assay using the full length human VEGF promoter F1–R3 and chimeric TEAD4 isoforms, TEAD4_216_ with a NLS and TEAD4_148_ without an NLS showed that the NLS domain was crucial for enhancers to function and that loss of the NLS converts a potent enhancer (TEAD4_148_) into an inhibitor. The chimeric TEAD4_216_+NLS isoform significantly enhanced promoter activity (p<0.01, n = 3).
(TIF)

Methods S1 **A description of electrophoretic mobility shift assays, culture of RF/6A cells, antibodies used and TEAD4 chimeric constructs.**
(DOC)
