# Supplementary figures and images for: The Related Transcriptional Enhancer Factor-1 Isoform, TEAD4216, Can Repress Vascular Endothelial Growth Factor Expression in Mammalian Cells
Source: PLoS One. 2012 Jun 22;7(6):e31260. doi: 10.1371/journal.pone.0031260 (PMC3382240; doi:10.1371/journal.pone.0031260)

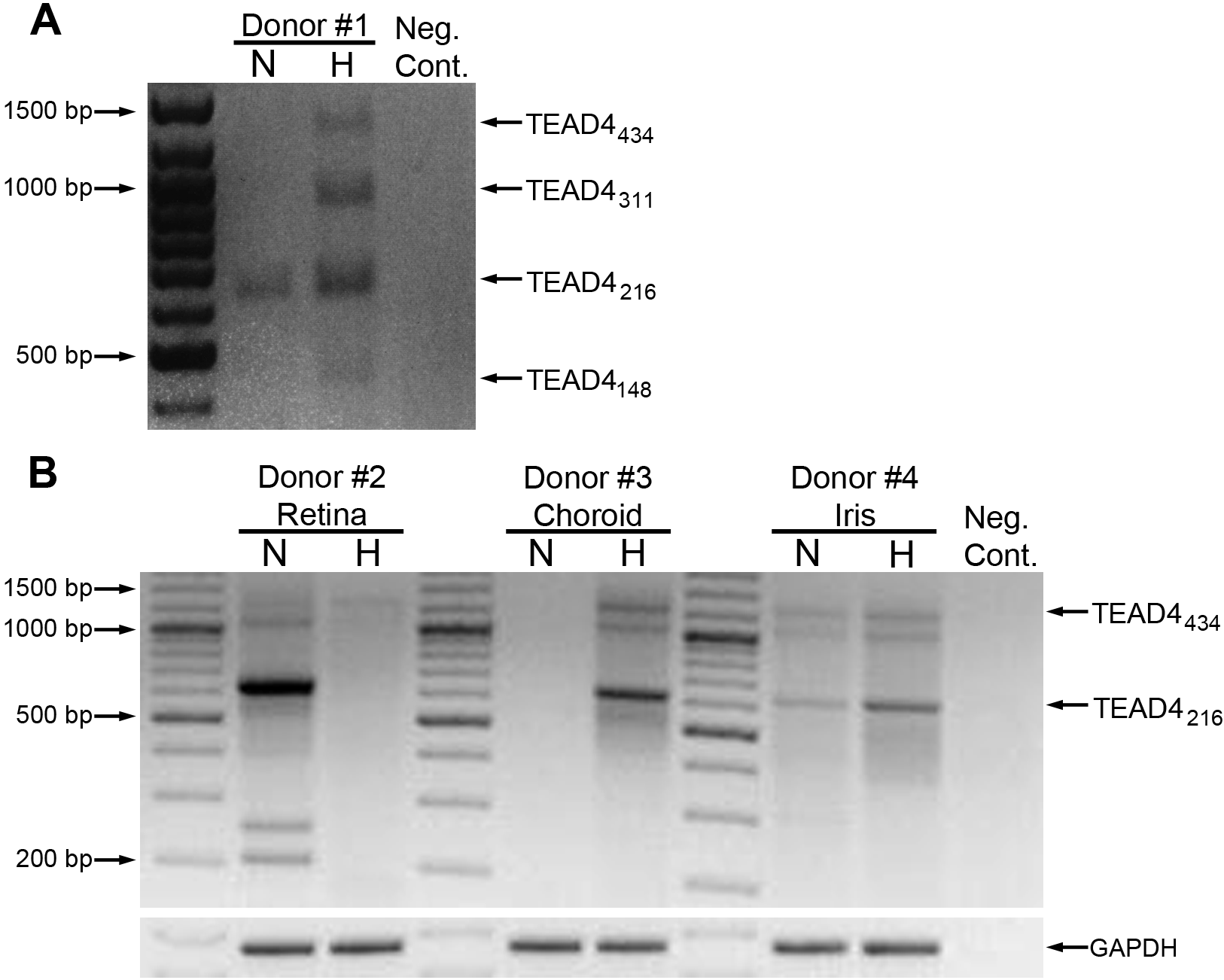

Supplement: Figure S1 — Human TEAD4 is differentially spliced within retinal, choroidal and iris derived vascular endothelial cells under hypoxia/normoxia. Agarose gel electrophoresis showing RT-PCR from RNA isolated from primary cultures of REC, CEC and IECs after culture under normoxic or hypoxic conditions. Donor #1 (pooled REC, CEC and IEC cells) gave products for TEAD4434 (1305 bp band), TEAD4311 (936 bp band), TEAD4216 (651 bp band) and TEAD4148 (447 bp band) under hypoxic conditions, and under normoxic conditions only the TEAD4216 product was amplified. TEAD4216 was present in REC, CEC and IEC but interestingly expression levels differed between each EC type and between normoxic and hypoxic conditions. (TIF) [file pone.0031260.s001.tif]

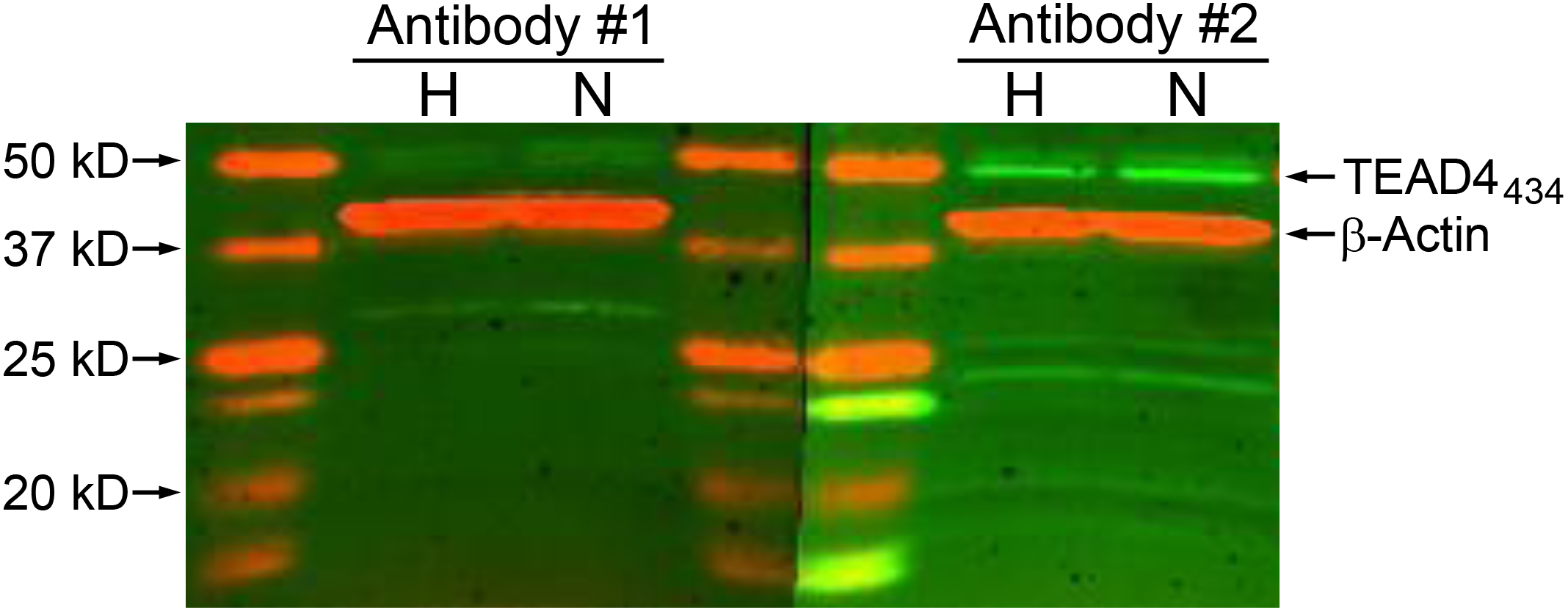

Supplement: Figure S2 — Western blot for TEAD4 on nonhuman primate ocular vascular endothelial cell proteins recognizes TEAD4434 and other isoforms. Two different commercially available antibodies (ARP33426 and ARP38276, Aviva) recognize the full length TEAD4 (∼50 kD) in monkey retinal-choroidal ECs (RF/6A). No significant difference in TEAD4 expression was observed when normoxic (N) samples were compared to hypoxia (H) treated samples. Other TEAD4 isoforms appear to also be recognized at 30 KD, 26 kD and 24 kD. (TIF) [file pone.0031260.s002.tif]

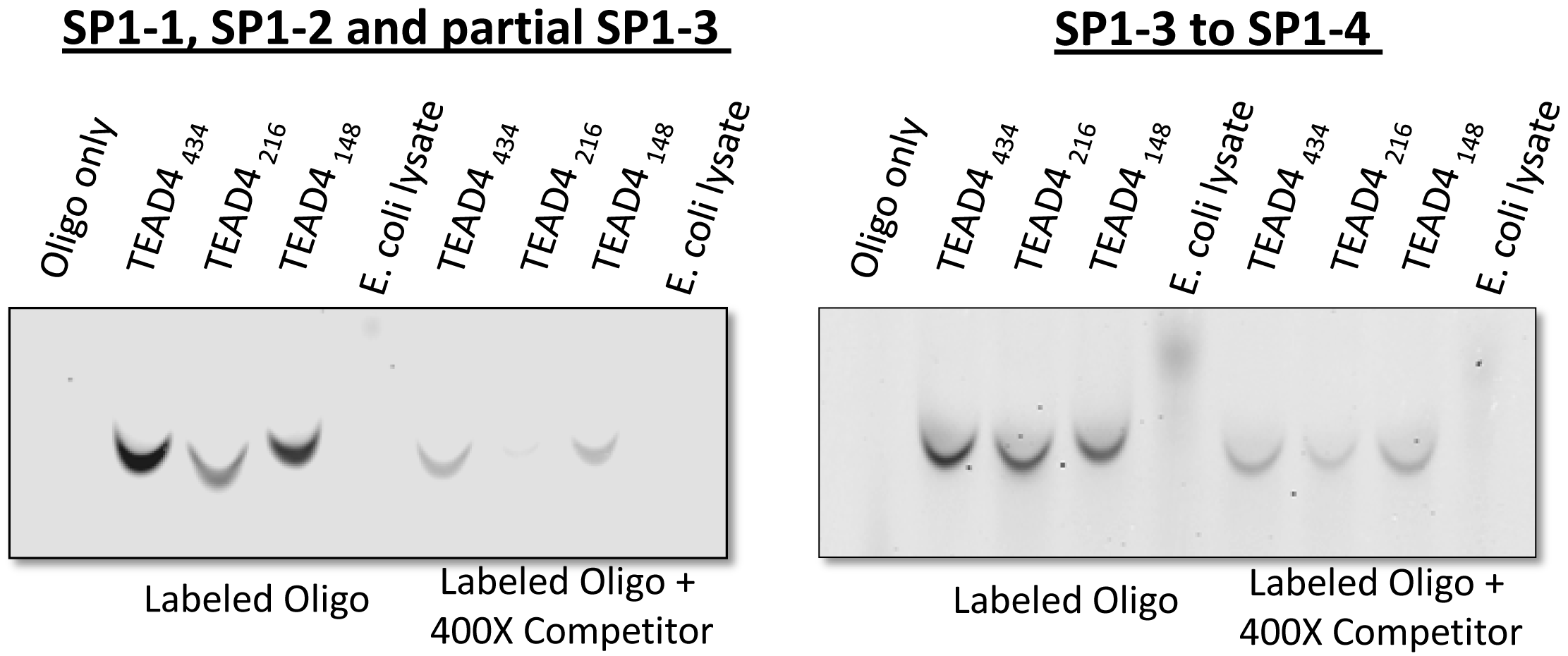

Supplement: Figure S3 — Band shift (EMSA) assay for TEAD4 isoforms on Sp-I sequences found within the VEGF promoter. The enhancer isoforms TEAD4434, TEAD4216 and TEAD4148 bound to the SpI sequences present within the human VEGF promoter. The repressor isoform TEAD4216, although lacking one of the 3 helixes present within the TEA DNA binding domain, is still able to bind the Sp-I sequences within the VEGF promoter. (TIF) [file pone.0031260.s003.tif]

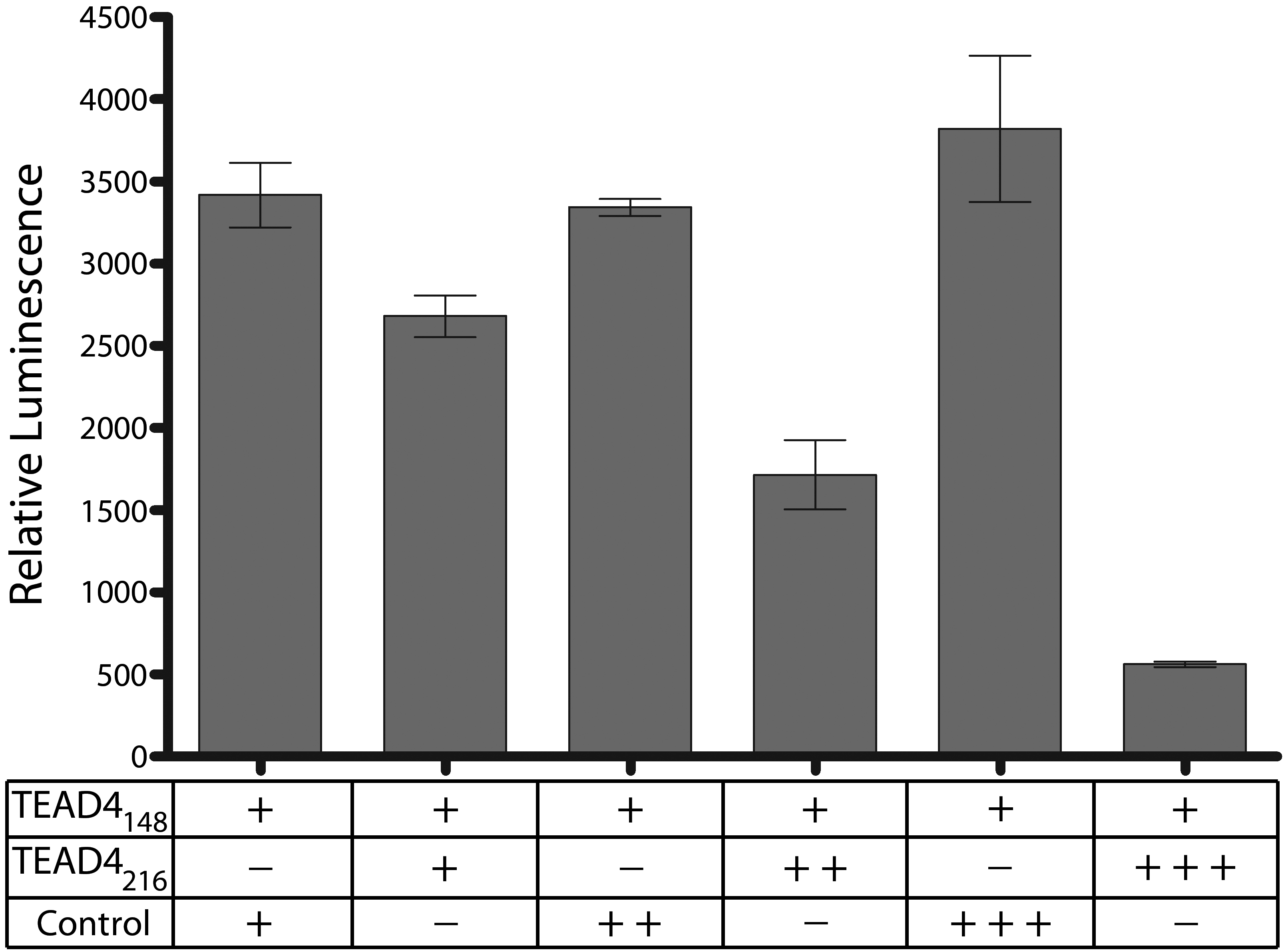

Supplement: Figure S4 — TEAD4216 can competitively inhibit enhancer mediated VEGF promoter activity. Increasing concentrations of a plasmid containing TEAD4216, inhibited the enhancer properties of TEAD4148. (TIF) [file pone.0031260.s004.tif]

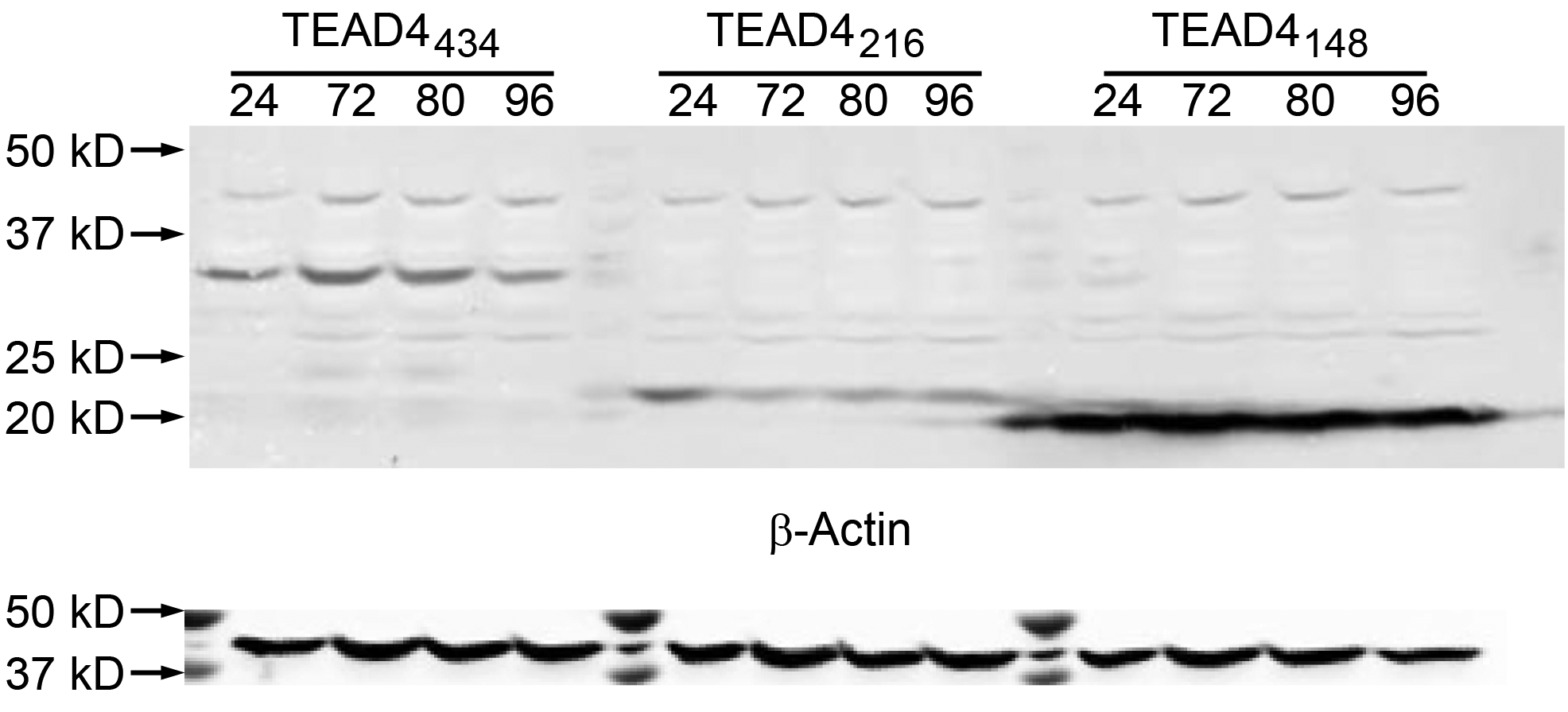

Supplement: Figure S5 — Expressed protein from transfected TEAD4 isoforms is stable over time. Protein from cells was isolated at various time points (24 to 96 hours) after transfection with pcDNA expression vector containing a TEAD4 isoform. Western blot was performed with a custom made TEAD4 antibody (Genemed Synthesis). (TIF) [file pone.0031260.s005.tif]

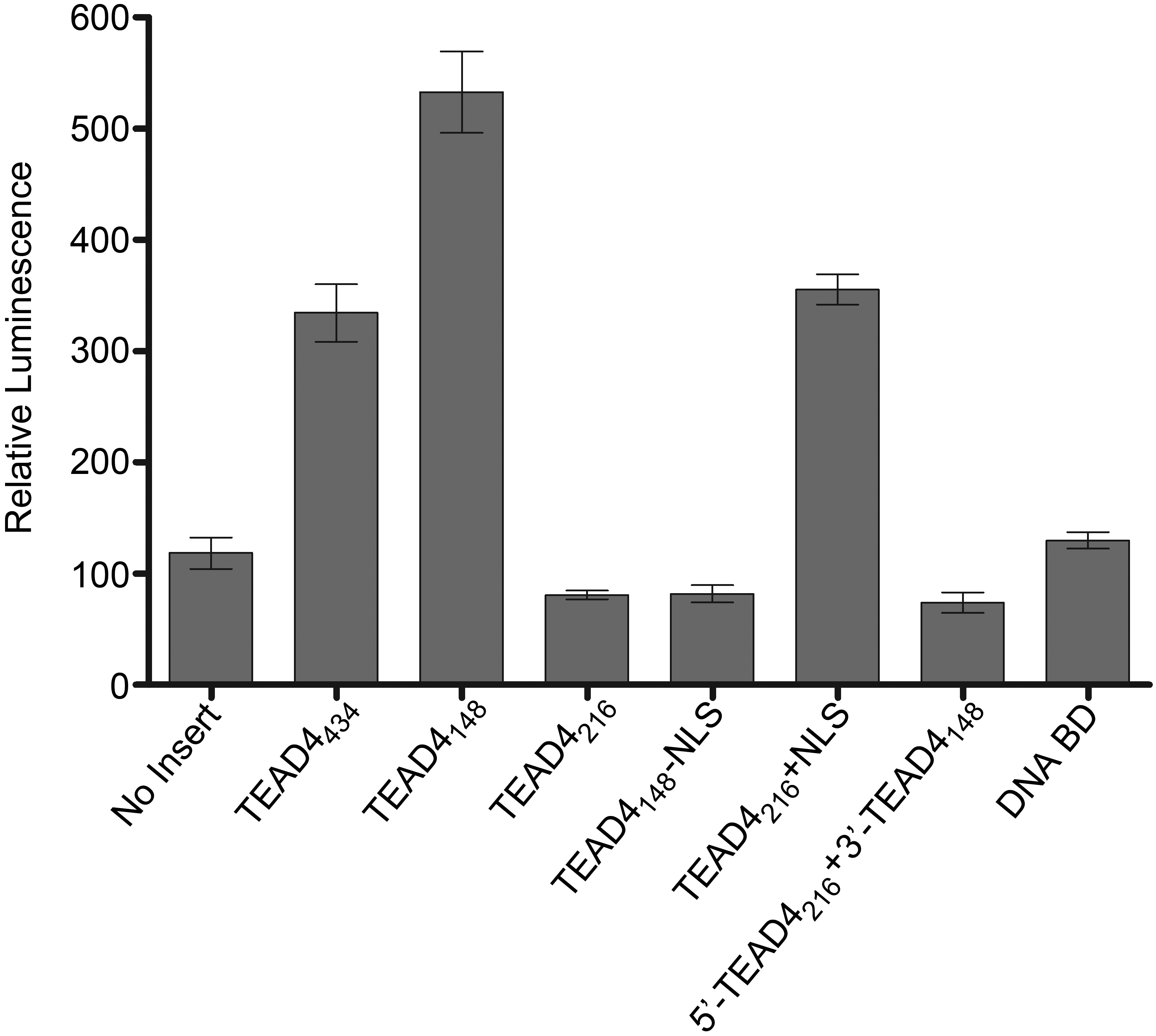

Supplement: Figure S6 — TEAD4216 requires an NLS to enhance VEGF promoter activity. A reporter assay using the full length human VEGF promoter F1–R3 and chimeric TEAD4 isoforms, TEAD4216 with a NLS and TEAD4148 without an NLS showed that the NLS domain was crucial for enhancers to function and that loss of the NLS converts a potent enhancer (TEAD4148) into an inhibitor. The chimeric TEAD4216+NLS isoform significantly enhanced promoter activity (p<0.01, n = 3). (TIF) [file pone.0031260.s006.tif]
