# Supplementary material for: The Related Transcriptional Enhancer Factor-1 Isoform, TEAD4216, Can Repress Vascular Endothelial Growth Factor Expression in Mammalian Cells
Source: PLoS One. 2012 Jun 22;7(6):e31260. doi: 10.1371/journal.pone.0031260 (PMC3382240; doi:10.1371/journal.pone.0031260)
Supplement: Methods S1 — A description of electrophoretic mobility shift assays, culture of RF/6A cells, antibodies used and TEAD4 chimeric constructs. (DOC) [file pone.0031260.s007.doc]

**Supplementary Materials and Methods:**

**Cultured cells:**

The rhesus macaque retinal-choroidal cells (RF/6A) were obtained from ATCC and cultured in Eagle’s minimum essential medium (EMEM) and 10% FBS.

**Electrophoretic mobility shift assay:**

Human TEAD4 isoforms were cloned into the pET-41 prokaryotic expression vector (Novagen) , transformed into Rosetta 2 *E. coli* cells (Novagen) and plated onto LB agar plates containing Kanamycin and Chloramphenicol. Single colonies were selected and seeded into 50ml flasks and grown for 3 hours at 37º C. IPTG was added (final concentration of 0.4mM) and cells grown for an additional 3 hours. Bacteria were pelleted and supernatant removed. Pellets were washed once with PBS. Total protein was isolated by adding 0.5ml RIPA buffer (150mM NaCl, 10mM Tris pH 7.2, 0.1% SDS, 1% triton X-100, 1% deoxycholate, 5mM EDTA) to the pellets, incubating on ice for 10 minutes and centrifuging at 15,000 rpm for 10 minutes. The supernatants were collected and assessed for protein concentration by a BCA assay.

Four Sp1 elements exist within human VEGF promoter (Sp1-1, Sp1-2, Sp1-3 and Sp1-4), situated in tandem within the first 100bp upstream of the VEGF transcription start site. The sequences spanning SpI-1 to SpI-4 were synthesized as two sets of complimentary single stranded oligos with an 800nm Infrared Dye (IR800) modification to the 5’ end. The first set was designed to contain Sp1-1, Sp1-2 and partial Sp1-3 (5’-aggctcgcctgtccccgccccccggggcgggc-3’), the second set was designed to contain Sp1-3 and Sp1-4 (5-gcgggccgggggcgg ggtcccggcggggcgga-3’). In addition to the IR800 labeled oligos, unlabeled oligos of the same sequence were also generated to act as a competitor to the protein/DNA binding reaction. Each set of complimentary oligos were mixed at equal concentrations and incubated at 100º C for 10 minutes and then allowed to cool to room temperature to anneal.

Each EMSA assay was performed by adding 2ul 10x binding buffer (100mM Tris, 500mM KCl, 10mM DTT; pH 7.5), 2ul of a 25mM DTT/2.5% Tween-20 mixture, 1ul of polydI/dC (1ug/ul in 10mM Tris, 1mM EDTA; pH 7.5), 1ul of 100mM MgCl2, 1ul of labeled annealed oligo (50nM) and 5ug of total protein. Binding reactions were mixed well and placed in a light tight drawer for 20 minutes. Competitor unlabeled oligo was used in half of the reactions at a 400X greater concentration then the labeled oligo. Reactions were loaded onto precast 5% TBE native acrylamide gels with the addition of 10X orange loading dye. Gels were run at 70V for 1 hour. Gels were immediately analyzed using the Licor Odyssey infrared scanner.

**Western blot analysis and antibodies**

Using 0.5ml RIPA buffer, total cellular protein was isolated from RF/6A and transfected 293T cells. Exactly 20ug of total protein with SDS-DTT loading dye was loaded for PAGE on a 4-20% Tris-Glycine gel (Lonza, Walkersville, MD). Proteins were transferred to nitrocellulose membrane, blocked with Odyssey blocking buffer, then probed with antibody, then rinsed with PBST and then probed with secondary antibody. The membrane was washed with PBST and then signals were visualized on the Licor Odyssey. Antibodies used: custom made rabbit anti-TEAD4 antibody (Genemed Synthesis, San Antonio, TX) at 1:5000 in Odyssey blocking buffer; goat anti-Beta actin at 1:5000; Rabbit anti-TEAD4 (Aviva #33426) at 1:1000, Rabbit anti-TEAD4 (Aviva #38276) at 1:4000 (Aviva Systems Biology, San Diego, CA). The custom TEAD4 antibody from Genemed Synthesis was raised to a 22 amino acid sequence (ITSNEWSSPTSPEGSTASGGSQ) present within exon 2 of human TEAD4 and detects all isoforms in this study. Aviva #38276 identifies TEAD4_434_, TEAD4_311_ and TEAD4_216_. Aviva #33426 detects TEAD4_434_ and TEAD_311_.

**Generation of chimeric TEAD4 clones**

Chimeric constructs of each TEAD4 isoforms were commercially synthesized (GenScript, Piscataway, NJ) and then subcloned into the pcDNA expression vector. The following amino acid numbering corresponds to the TEAD4_434_ sequence of 434 amino acids.

TEAD4_434_ = 1 to Glu-434 aa;

TEAD4_216_ = 1 to Thr-92 +Ser-311 to Glu-434;

TEAD4_148_ = 1 to Lys-118 + Phe-162 to Gln-182 + His-426 to Glu-434;

TEAD4_216_+NLS = 1 to Lys-118 + Ser-311 to Glu-434;

TEAD4_148_-NLS = 1 to Lys-118 (with the NLS sequence from Val-104 to Ala-110, replaced with 7x His amino acids) + Phe-162 to Gln-182 + His-426 to Glu-434;

5’-TEAD4_216_+ 3’-TEAD4_148_ = 1 to Thr-92 + Phe-162 to Gln-182 + His-426 to Glu-434;

DNA BD = 1 to Lys-118.
